# Circadian regulation of translation

**DOI:** 10.1080/15476286.2024.2408524

**Published:** 2024-09-26

**Authors:** Jiali Lyu, Yanrong Zhuang, Yi Lin

**Affiliations:** State Key Laboratory of Membrane Biology, IDG/McGovern Institute for Brain Research, Tsinghua-Peking Joint Centre for Life Sciences, School of Life Sciences, Tsinghua University, Beijing, China

**Keywords:** Circadian rhythm, translation, translation regulation, phase separation, stress

## Abstract

Most, if not all organisms exhibit robust rhythmicity of their biological functions, allowing a perpetual adaptation to external clues within the daily 24 hours-cycle. Studies on circadian rhythm regulation primarily focused on transcriptional level, considering mRNA levels to represent the primary determinant of oscillations of intracellular protein levels. However, a plethora of emerging evidence suggests that post-transcriptional regulation, particularly rhythmic mRNA translation, is not solely reliant on the oscillation of transcription. Instead, the circadian regulation of mRNA translation plays a critical role as well. A comprehensive understanding of these mechanisms underlying rhythmic translation and its regulation should bridge the gap in rhythm regulation beyond RNA fluctuations in research, and greatly enhance our comprehension of rhythm generation and maintenance. In this review, we summarize the major mechanisms of circadian regulation of translation, including regulation of translation initiation, elongation, and the alteration in rhythmic translation to external stresses, such as endoplasmic reticulum (ER) stress and ageing. We also illuminate the complex interplay between phase separation and mRNA translation. Together, we have summarized various facets of mRNA translation in circadian regulation, to set on forthcoming studies into the intricate regulatory mechanisms underpinning circadian rhythms and their implications for associated disorders.

## 1. Introduction

In the delicate dance of life on Earth, an unseen conductor orchestrates a symphony of biological rhythms, guiding the activities of organisms in harmony with the planet’s daily rotation. These biological clocks regulate a plethora of physiological activities, including body temperature, blood pressure, as well as sleep-wake patterns. In mammals, the circadian system includes a central pacemaker, the suprachiasmatic nucleus (SCN), located in the hypothalamus, as well as other peripheral oscillators in various tissues. The SCN serves as the central hub that generates and transmits clock’s signals to peripheral organs. Intriguingly, while peripheral oscillators synchronize with the central clock, they act relatively independently, suggesting an age-old mechanism encoded in the genes for circadian rhythms. Disruptions in circadian rhythms are linked to diseases such as cancer, type 2 diabetes, psychiatric disorders, and neurodegeneration [1].

At the molecular level, circadian rhythms are primarily regulated by a negative transcription-translation feedback loop (TTFL). In mammals, CLOCK, BMAL1, Period (PER), and Cryptochrome (CRY) are involved in this TTFL as four major components. In response to various external clues, such as light or metabolic signals, the two transcription factors CLOCK and BMAL1, form heterodimers and bind to E-box sequences in the promoters of PER, CRY, and other clock-related genes, thus activating their transcription. The transcribed mRNAs are then translated outside the nucleus. Subsequently, PER and CRY proteins form heterodimers in the cytoplasm and are transported back into the nucleus where they inhibit the transcriptional regulation mediated by CLOCK and BMAL1. This intricate negative feedback loop ensures precise regulation of circadian rhythms while maintaining a 24-hour cycle [2].

These rhythmic transcriptional processes ensure the oscillation of core clock gene expression, thereby conveying clock information to downstream pathways – an elemental intracellular regulatory paradigm. Extensive research has been dedicated to identifying mRNAs that exhibit rhythmic changes in abundance across different cells and tissues throughout the day. These insights clearly emphasize the crucial role of rhythmic transcription in generating oscillatory dynamics of gene expression across various organisms and cellular contexts.

However, the expression of clock-related genes in mouse livers is also determined by post-transcriptional mechanisms. Observations indicate that over 25% of the rhythmic mRNA accumulation originates from genes that do not exhibit oscillatory behaviour at the transcription level [3–5]. Simultaneously, proteomics studies also suggest that ~ 10% of rhythmic protein expression displays no corresponding fluctuations in their mRNA counterparts in mouse livers [6,7]. The lack of concordance between the transcriptome and proteome likely reflects the significant contributions of post-transcriptional regulation, including mRNA processing, mRNA stabilization, protein synthesis, and protein degradation. These processes may inherently harbour components contingent upon rhythmicity.

mRNA translation, situated at the terminus of transcriptional events, necessitates a comprehensive regulatory framework affecting mRNA stability, translation efficiency, and protein quality control. Given the pivotal role of mRNA in the overall translational process, research on circadian regulation by post-transcriptional mechanisms has predominantly focused on directly studying mRNA processing and its stability [8]. However, a substantial subset of components involved in the assembly of the translation machinery exhibits rhythmic fluctuations, indicating the importance of protein synthesis downstream from circadian rhythm output [9,10]. Translation is crucial in integrating genetically encoded rhythmic signals with various external or internal stimuli, including endoplasmic reticulum (ER) stress and ageing. Most of the translation-related factors are not only governed by the upstream transcription rhythm, thereby exhibiting rhythmic expression, but also regulated by downstream processes such as ribosome biosynthesis, initiation machinery assembly, and other factors involved in rhythmic translation.

In this review, we discuss our current understanding of how translation contributes to the generation, regulation, and maintenance of circadian rhythms. In addition, we assess the impact of phase separation and various stresses on rhythmic translation, thus highlighting the importance of translational regulation within the intricate framework of circadian clocks.

## 2. Circadian regulation at the translation initiation step

Initiation of translation is subject to precise regulation by circadian rhythms, exerting influence over multiple aspects such as ribosome biogenesis and the assembly of translation machinery.

Circadian rhythms regulate these processes at multiple stages. Firstly, during ribosome biogenesis, both the transcription of ribosomal RNA (rRNA) and the synthesis of ribosomal proteins oscillate along the circadian rhythm.

**Figure 1.**
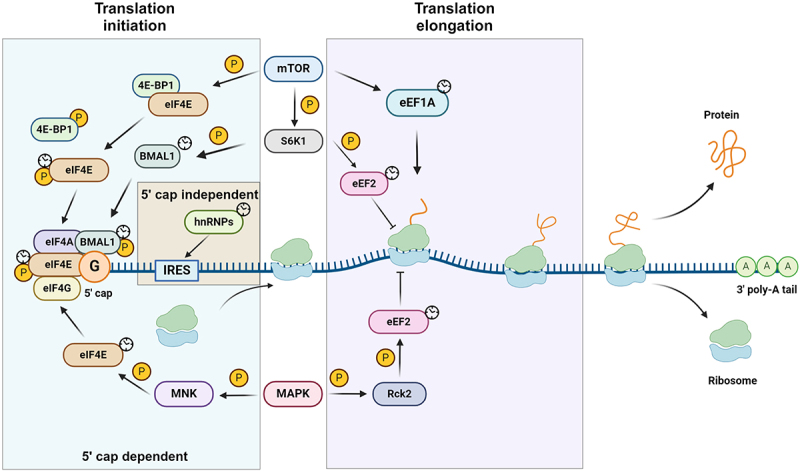
The regulation mechanism of translation initiation and elongation in circadian rhythms. Translation initiation occurs through 5' cap-dependent and 5' cap-independent patterns. The mammalian or mechanistic target of rapamycin (mTOR) and mitogen-activated protein kinases (MAPK) pathways control 5' cap-dependent initiation through rhythmic expression and phosphorylation of downstream proteins. Moreover, heterogeneous nuclear ribonucleoproteins (hnRNPs) regulate the 5' cap-independent translation initiation through rhythmic interactions with IRES elements. For translation elongation, mTOR and MAPK regulate the rhythmic expression and phosphorylation of eukaryotic translation elongation factors, such as eEF1A and eEF2 (created with BioRender).

Subsequently, the assembly of translation machinery occurs through two major pathways: 5' cap-dependent translation and 5' cap-independent translation. Both of these two processes show robust rhythms in line with circadian clocks. The 5' cap-dependent translation relies on recognition of the 5' cap of mRNA by eukaryotic initiation factors (eIFs), including eIF4E, eIF4G, and eIF4A, all of which form the eIF4F complex, followed by the recruitment of the 43S pre-initiation complex as well as the 60S ribosomal subunit [11]. During the process of assembly, the expression and post-translational modifications of certain eukaryotic initiation factors exhibit rhythmic oscillation. Alternatively, when the 5' cap-dependent translation fails upon cellular stress and viral infection, a 5' cap-independent mechanism is employed to initiate translation via the internal ribosome entry site (IRES). The majority of IRES recruit ribosomes for translation initiation through interacting with IRES *trans-*acting factors (ITAFs) [12]. Specific sets of ITAFs oscillate in protein expression and nucleoplasmic shuttles to sustain rhythmic interactions with IRES, thus controlling the rhythm of translation (Figure 1).

Here, we delve into the rhythmic regulation of translation initiation, acknowledging that numerous additional pathways contribute to this intricate process.

### 2.1. Circadian regulation of ribosome biogenesis

**Figure 2.**
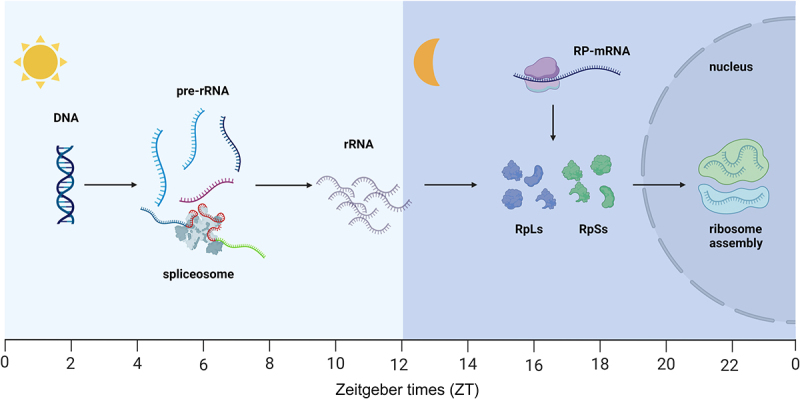
Ribosome biogenesis events occur sequentially at different times of the day in mouse livers. Pre-rRNA transcription and processing peaked at ZT6. Ribosomal proteins translation occurs around ZT18. After that, newly assembled ribosomes enter the nucleus in a large number near ZT22. The zeitgeber times (ZT), with ZT0, lights on; ZT12, lights off (created with BioRender).

Ribosome biogenesis involves the assembly and maturation of ribosomal RNA and ribosomal proteins. The sequential process of ribosome biogenesis occurs at different zeitgeber times (ZT) of the day [13] (Figure 2). In mouse livers, the expression level of rRNA transcription machinery, including the RNA polymerase I subunit, peaks at ZT6 and facilitates the transcription of pre-rRNA. Concurrently, the activity of protein complexes responsible for rRNA processing and pre-ribosome assembly also reaches its zenith. Following the synthesis and maturation of rRNA during the daytime, the mRNA translation of ribosomal protein takes place around ZT18. The final phase of ribosome assembly involves the rhythmic entry of the pre-60S ribosome into the nucleus, peaking near ZT22 [13,14]. The rhythms of expression levels of rRNA and ribosomal proteins are under the regulation of upstream clock proteins. In the absence of core clock proteins in TTFL, such as CRY1/2 or BMAL1, the rhythmic ribosome biogenesis is disrupted [14]. On the other hand, the upstream binding factor 1 (UBF1), responsible for establishing and maintaining an active chromatin state, is rhythmically expressed at both mRNA and protein levels in mouse livers. Lack of UBF1 also induced rhythm disorders in ribosome biogenesis [14].

In addition to those found in mammals, robust circadian rhythms in ribosome biogenesis have also been observed in plants and insects. In *Arabidopsis*, the translation of ribosomal proteins reaches its peak at night, and overexpression of the clock gene CCA1 showed phase shifts in the peak of translation, with an approximately 6 h delay [15]. Similarly, rhythmic transcription of many ribosomal proteins has been documented in *Drosophila*, encompassing crucial components of small and large ribosomal subunits [16]. This evidence underscores the highly conserved nature of the rhythm of ribosome biogenesis across phyla.

### 2.2. 5' cap-dependent translation initiation

In 5' cap-dependent mRNA translation initiation, the rate-limiting step is the binding of eIFs to the 5' cap, a process primarily regulated by circadian rhythms via three signalling pathways: the mammalian or mechanistic target of rapamycin (mTOR), mitogen-activated protein kinases (MAPK) [17], and integrated stress response (ISR) pathways [18] (Figure 1).

mTOR, a critical regulator of cellular metabolism, orchestrates the rhythmic protein synthesis by phosphorylating the translation initiation factors following a rhythmic pattern. The mTORC1 complex specifically phosphorylates 4E-BP, enabling its dissociation from eIF4E and facilitating the subsequent binding of eIF4E to eIF4G. This binding triggers the recognition of the 5' cap of mRNA by the translation initiation complex, ultimately leading to the initiation of protein synthesis. Given the rhythmic oscillation in mTOR activity, 4E-BP undergoes rhythmic phosphorylation by mTOR [19]. In the mouse suprachiasmatic nucleus (SCN), the level of phosphorylated 4E-BP1 peaks during daylight hours. This diurnal rhythm modulates the translation initiation of vasoactive intestinal peptide (Vip) mRNA, which is intricately linked to PER2 production and circadian function. Moreover, mTOR also phosphorylates S6K, subsequently regulating the phosphorylation status of RpS6, eEF2K, PDCD4, and eIF4B. PDCD4 and eIF4B, in turn, modulate the level and activity of eIF4A, thereby exerting control over translation initiation [18].

Notably, RpS6, as a downstream target of mTOR signalling, exhibits diurnal fluctuations in its phosphorylation levels. Phosphorylated RpS6 proteins reach their peak levels during daylight hours and trough levels during night-time. In addition, studies in the mouse hippocampus reveal a rhythmic pattern of phosphorylation and activation for a series of translation initiation proteins, including eIF4E, 4E-BP, RpS6, and the eIF4F cap-complex, during the day. Conversely, in the mouse liver, eIF4E is primarily phosphorylated during daylight hours, while eIF4G, eIF4B, 4E-BP1, and RpS6 are phosphorylated primarily during the night [14].

mTOR, not only regulates translation initiation factors, but also plays a pivotal role in modulating translation initiation directly, specifically through phosphorylating the core clock gene BMAL1^20^. In mouse livers, BMAL1 serves as a substrate for S6K1, undergoing rhythmic phosphorylation. This phosphorylated BMAL1 interacts with the translation initiation complex, thereby influencing protein synthesis [20]. Notably, the level of phosphorylated BMAL1 peaks during the night and declines during the daytime, aligning closely with the rhythmic pattern of ribosome biogenesis [14]. This coordinated regulation of BMAL1 phosphorylation by mTOR further underscores the intricate network of pathways that mTOR oversees in controlling translation initiation. Together, these observations suggest that the circadian rhythm controls translation initiation primarily through the mTOR signalling pathway [21].

On the other hand, within the MAPK pathway, the eIF4E is phosphorylated by MAPK through the downstream MNK protein kinase, regulating the assembly of the translation initiation complex [18,22], and the rhythm of eIF4E phosphorylation displays significant phase differences in different brain regions [23]. In mammals, the SCN exhibits distinct phosphorylation patterns of MAPK across different regions. In the ventrolateral region of the mouse SCN, MAPK phosphorylation peaks at night, while in the dorsomedial region, it reaches a brief peak in the early day [24]. Upon light exposure, MAPK is activated, triggering the phosphorylation of MNK and subsequently eIF4E at Ser209. Furthermore, in the mouse SCN and hypothalamus, this rhythmic phosphorylation of eIF4E regulates the translation initiation of PER1 and PER2, maintaining the oscillation of PER proteins and thus sustaining circadian rhythms [19].

Moreover, the ISR pathway, which mediates neurons’ response to various stress stimuli to maintain cellular homoeostasis, has been shown to regulate the circadian rhythm in mice. At the core of this process is the phosphorylation of eIF2α, which is crucial for delivering methionyl-tRNA-Met to small ribosomal subunits to form the 43S pre-initiation complex [25]. When cellular stress activates the ISR signalling pathway, eIF2α kinase GCN2 rhythmically phosphorylates eIF2α in the mouse suprachiasmatic nucleus (SCN). This rhythmic phosphorylation of eIF2α subsequently regulates the rhythmic translation of the transcription factor ATF4. The resulting rhythmic ATF4 proteins typically activate the transcription of PER2, thereby sustaining circadian rhythms within the SCN [26].

### 2.3. 5' cap-independent translation initiation

The role of 5' cap-independent translation initiation is pivotal. IRES elements in mRNA molecules recruit ribosomes and initiate 5' cap-independent translation, under the assistance of ITAFs. ITAFs rhythmically interact with IRES, thus regulating the rhythmic nature of translation (Figure 1). In the past, the 5' cap-independent translation was regarded as a compensatory pathway for 5' cap-dependent translation. While many mRNAs containing IRES undergo 5' cap-dependent translation, they switch to IRES-mediated translation initiation when cells are under stress [11].

Most IRES elements require the binding of ITAFs, which shuttle between the nucleus and cytoplasm [27]. A well-studied ITAF is the polypyrimidine tract-binding protein, also known as hnRNP I, which promotes cellular IRES activity. Interestingly, the expression level of this protein is unchanged, but it can rhythmically interact with IRES through periodic nucleoplasmic shuttling [28].

To date, an increasing number of ITAFS have been identified as hnRNPs, which actively regulating IRES-mediated translation initiation in accordance with circadian rhythms. For instance, hnRNP Q and hnRNP I exhibit rhythmic binding to the IRES in the 5' UTR of Rev-erb alpha mRNA, thereby enhancing its rhythmic translation [28]. Furthermore, the expression level of hnRNP Q demonstrates a rhythmic pattern, peaking during the night in rat pinealocytes. This protein also binds to the IRES of AANAT mRNA, facilitating its rhythmic translation and consequently regulating the circadian rhythm of melatonin production [29]. hnRNP A1 rhythmically moves between the nucleus and cytoplasm, interacting with the IRES of Nfil3 mRNA to enhance its translation, thereby sustaining the continuous oscillation of Nfil3 protein levels [30].

In essence, the oscillatory nature of translation initiation is orchestrated through various regulatory layers, ensuring a periodic initiation synchronized with the circadian rhythm. Ribosome biogenesis involves rhythmic expression of rRNAs and ribosomal proteins. In canonical 5' cap-dependent translation, essential components of the translation machinery, including eIF4E and BMAL1 proteins, exhibit rhythmic expression and phosphorylation. Similarly, in 5' cap-independent translation, the translation rhythm is sustained through oscillating interactions between ITAFs and IRES elements. This complex regulation ensures that translation initiation is temporally coordinated, vital for subsequent steps in maintaining cellular homoeostasis and biological functions.

## 3. Circadian regulation at the elongation step

In addition to translation initiation, compelling evidence indicates that circadian rhythms have a regulatory impact on translation elongation, thus influencing the rhythmic oscillation of overall protein synthesis. The translation elongation process encompasses repeated decoding, peptidyl transfer, and tRNA_2_-mRNA translocation. During elongation, elongation factor eEF1A is recruited to the translation machinery and transports aminoacyl-tRNA (aa-tRNA) to the A-site of the 80S ribosome. Furthermore, eEF2 facilitates the translocation of the tRNA_2_-mRNA complex on the ribosome to the next codon, resulting in the sustainability of translation elongation [31]. The expression and modification of eEF1A and eEF2 are tightly controlled, given their pivotal roles as major regulators in the elongation process (Figure 1).

The major translation elongation factors show a predominant rhythmic expression. Additionally, the mTOR pathway, MAPK pathway, and additional control factors regulate the expression and modification of translation elongation factors, thus modulating the rhythmicity of the translation process. Proteomic analysis in mouse livers showed that the protein levels of eEF1A and eEF2 undergo fluctuations along the circadian clock, displaying a peak similar to that of translation initiation factors around mid-night [32]. In addition to the rhythmic expression of eEF1A, which is subject to regulation by the mTOR pathway [33], and it is possible that polyamines may also contribute to its circadian regulation.

### 3.1. The regulation of translation elongation by mTOR pathway

The involvement of mTOR in the regulation of rhythmic translation extends beyond translation initiation and exhibits a pivotal role in translation elongation [34]. For example, in mouse SCN, eEF1A translation is augmented by p70 S6 kinase following light-induced activation of the mTOR signalling pathway [35]. This pattern aligns with light-induced S6 phosphorylation, suggesting that light-dependent circadian rhythms are intricately linked to the regulation of translation elongation. However, whether the phosphorylation of eEF1A regulates circadian rhythms by modulating translation elongation remains uncertain.

Moreover, eEF2 phosphorylation can be negatively regulated by mTOR signals in mouse [36]. Activated eEF2K is deemed essential for inducing eEF2 phosphorylation [36]. Considering that S6K1 in mTORC1 inactivates eEF2K [37] and the temporal regulation of mRNA translation is rhythmically coordinated by the mTOR pathway [17], it can be hypothesized that the rhythmic translation elongation mediated by mTOR signalling in mammals is conservatively driven by eEF2K inactivation. Notably, rapamycin, an mTOR inhibitor, did not affect eEF1A transcription under stimulus, implying that the mTOR pathway induces eEF1A expression through a translation-dependent mechanism during the daytime [33].

While these findings provide important insights, the precise mechanisms and pathways connecting mTOR to protein elongation remain incompletely understood. Further research is needed to elucidate the complex interplay between mTOR signalling and the regulation of translation elongation.

### 3.2. The regulation of translation elongation by MAPK pathway

In mice, the circadian clock triggers a daily cycle in the p38 osmosensing (OS) MAPK pathway, resulting in rhythmic phosphorylation and activation of MAPK-activated protein kinase Rck2. This, in turn, leads to rhythmic phosphorylation of eEF2, suppressing protein elongation of nearly all mRNAs by disrupting its binding to the ribosome [34,36]. The phosphorylation rhythm of eEF2 has been found not only in mammals, but also in plants. In *Neurospora crassa*, the phosphorylation cycle of FRQ and its interaction with WCC are central features of the circadian rhythm, ensuring precise and robust oscillations. WCC directly controls the transcription of gene *os-4*, which encodes the mitogen-activated protein kinase kinase kinase (MAPKKK). Rhythmic transcription of *os-4* leads to a cascade of phosphorylation reactions that orchestrates the accumulation of phosphorylated eEF2. Notably, eEF2 phosphorylation peaks during the subjective morning when translation elongation is predominantly suppressed [38].

Together, the MAPK pathway manipulates hierarchical phosphorylation to regulate the level of phosphorylated eEF2, one of the key factors in translation elongation. However, additional investigations of MAPK are imperative to unveil further mechanisms governing the rhythmic elongation step.

### 3.3. The regulation of translation elongation by polyamines

Polyamines, ubiquitously present in terrestrial organisms, stimulate the translation of eEF1A [39]. Polyamine levels oscillate in a rhythmic pattern, presumably under the regulatory control of the BMAL1-CLOCK complex. Intracellular polyamines regulate the circadian rhythm in cells by impacting the interaction between the circadian repressors PER2 and CRY1 [40]. However, at present it remains unclear how oscillating polyamine levels contribute to the rhythmic translation of eEF1A. More direct evidence is required to substantiate this hypothesis.

To summarize, the rhythmic expression of eEF1A and phosphorylation of eEF2, are under the tight regulation of the mTOR pathway, leading to rhythmic oscillations in translation elongation processes. Moreover, eEF2 phosphorylation also undergoes regulation by the MAPK pathway. The expression of EF1A may be regulated by rhythmic polyamines. These diverse ways ensure the precise regulation of translation elongation, and thus contributing to the rhythm of translation.

## 4. The regulatory role of phase separation in rhythmic translation

Beyond the traditional regulation via direct specific interactions among molecules and pathways, phase separation has recently emerged as a novel regulatory element of rhythmic translation. Phase separation refers to the process in which biological macromolecules (such as proteins, DNA, and RNA) in a solution, under certain conditions of temperature, pH, and salt concentration, form distinct concentrated and dilute phases (or multiple phases) when their concentration reaches a critical threshold. In the cellular context, this phase separation phenomenon is viewed as an important mechanism driving the formation of membrane-less organelles within cells [41].

A prime example is the assembly of mRNA and RNA-binding proteins into ribonucleoprotein (RNP) granules in the cytoplasm. These granules, once believed to be solely involved in mRNA storage and translation inhibition, are now understood to play a pivotal role in translation activation [42]. Furthermore, later studies have demonstrated that phase separation plays a regulatory role in translation during circadian rhythm processes [43].

The advantage of phase separation lies in its flexibility in regulating dynamic changes in translation. Membrane-less organelles formed through phase separation, due to their lack of membrane coating, exhibit enhanced dynamics. This allows the phase separation system to swiftly respond to diverse intracellular signals, such as translation activation, by concentrating a significant number of ribosomes, translation-related proteins, and mRNAs, enabling a rapid and efficient translation process.

**Figure 3.**
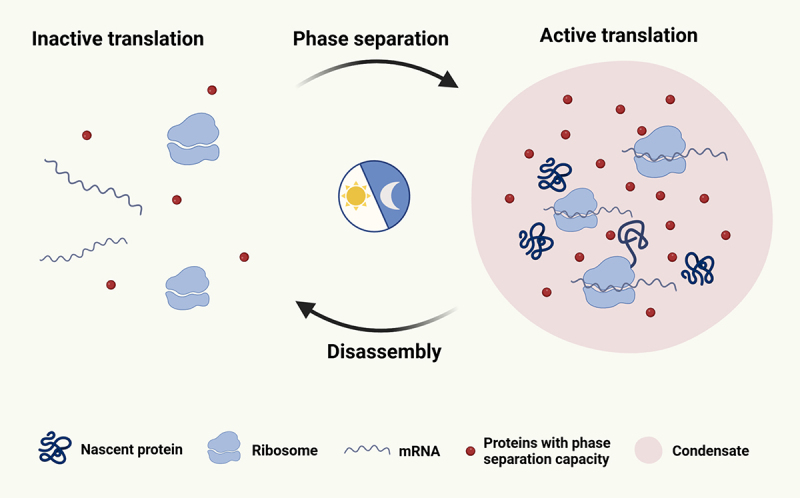
The formation and dissipation of protein condensates regulate the rhythm of translation. When phase separation does not occur, ribosomes and mRNAs are dispersed, resulting in inactive translation. When phase separation occurs in response to intracellular signals, condensates are quickly assembled to recruit ribosomes, mRNAs and translation factors to activate translation initiation (created with BioRender).

Furthermore, this dynamic characteristic confers evident advantages in investigating circadian rhythm oscillations regulated by intricate environmental factors and intracellular signals. Next, more detailed examples should be presented to exhibit the regulatory role of phase separation in circadian regulation, especially rhythmic translation (Figure 3).

Certain RNA-binding proteins undergo phase separation, resulting in the formation of dynamic protein condensates [44]. These condensates exhibit rhythmic behaviour and serve as regulatory centres that coordinate translational oscillations. For example, a study in *Neurospora crassa* showed that the small nuclear ribonucleoprotein 1 (SNR-1), a component of cytoplasmic messenger ribonucleoprotein granules, undergoes rhythmic phase separation [38]. In that study, the resulting condensates exhibited temporal recruitment of *zip-1* mRNAs, thereby facilitating translation through eIF2α in a rhythmic manner [38].

Furthermore, the RNA-binding protein Ataxin-2 has emerged as a potential key player in circadian regulation of translation, specifically in PER protein synthesis in *Drosophila* and mammals [43,45]. Recent investigations have uncovered that Ataxin-2 and its homologs dynamically undergo phase separation, forming spatiotemporal condensates throughout the circadian cycle. These condensates exhibit a strong binding affinity for the 3' UTR of PER2 mRNAs, actively recruiting translation machinery to promote rhythmic translation of PER2. Deletion of Ataxin-2 and Ataxin-2-like proteins disrupts this translation cycle, leading to weakened circadian rhythms in murine models [43]. This intricate interplay between phase-separated condensates and translational regulation underscores their pivotal role in controlling rhythmic biological processes.

An additional example of phase separation involves the Fragile X-associated protein (FXR) in mice, which is renowned for its propensity to undergo phase separation. Specifically, FXR is hypothesized to potentially contribute to circadian translational regulation. Among the Fragile X-associated protein family, F×R1 is highly expressed in late spermatids, where it undergoes phase separation to form condensates that serve as storage sites for various mRNAs. Following condensation, the translation machinery is recruited to activate the translation of the stored mRNAs, initiating the spermiogenesis process in a precisely defined spatial and temporal manner [46]. Other studies performed in mouse livers indicated that FXR also participates in the regulation of circadian rhythm. Critically, the disruption of FXR1/2 led to disturbances in rhythmic proteins, including PER1/2 and CRY1 [47], and the FXR protein family was found to enhance BMAL1-NPAS2 transcriptional activity mediated by PER1/2 [47]. This suggests that phase-separated F×R1 might regulate the translation of rhythm genes, such as PER, thereby exerting control over circadian rhythms.

Current studies on translation activation through phase separation predominantly centre around the initiation stage of translation. This emphasis can be attributed to two main factors. Firstly, the recruitment of vital biological macromolecules, including ribosomes, translation-related proteins, RNA-binding proteins, and mRNA, during initiation is a rate-limiting step in translation. Secondly, the dynamic properties of phase separation and its ability to concentrate molecules make it well-suited for facilitating enrichment processes that involve a large number of molecules.

Future investigations should focus on the potential of various condensates to selectively initiate mRNA translation during circadian rhythms. In addition to translation initiation, it will be equally important to explore whether the process of translation elongation is governed by phase separation, as well as how the subcellular compartmentalization is integrated upon various environmental cues, such as light and feeding, thus figuring out the specific role of phase separation in the circadian regulation of translation.

## 5. Rhythmic translational regulation in response to stress

We have discussed how organisms maintain translation rhythms in normal conditions, but how do organisms maintain rhythms in the face of cellular stress? When cell experience various stresses, such as endoplasmic reticulum (ER) stress, oxidative stress, nutrition starvation, and viral infection, the preservation of routine translation machinery in cells could be detrimental [48]. Importantly, these stresses may also alter the period and amplitude of cellular rhythm [49]. Protein synthesis and folding take place within the endoplasmic reticulum (ER), making ER stress essential for sustaining overall protein synthesis and maintaining protein homoeostasis. Concurrently, ageing, as a chronic stressor, disrupts the rhythmic process of mRNA translation. We now present a comprehensive overview of the influence of these two types of stress, ER stress and ageing, on rhythmic mRNA translation. Next, we’ll look at a few examples to explain rhythmic translational regulation under stress in detail.

### 5.1. Circadian regulation of translational processes during Endoplasmic Reticulum (ER) stress

Numerous studies have investigated the mechanism underlying ER stress-mediated translational regulation [50] and the interplay between ER stress and circadian rhythm [51], suggesting that these processes are intricately linked. Extensive disruptions in cells can compromise protein folding efficiency in the ER, leading to the accumulation of misfolded proteins, known as ‘ER stress’ [52].

**Figure 4.**
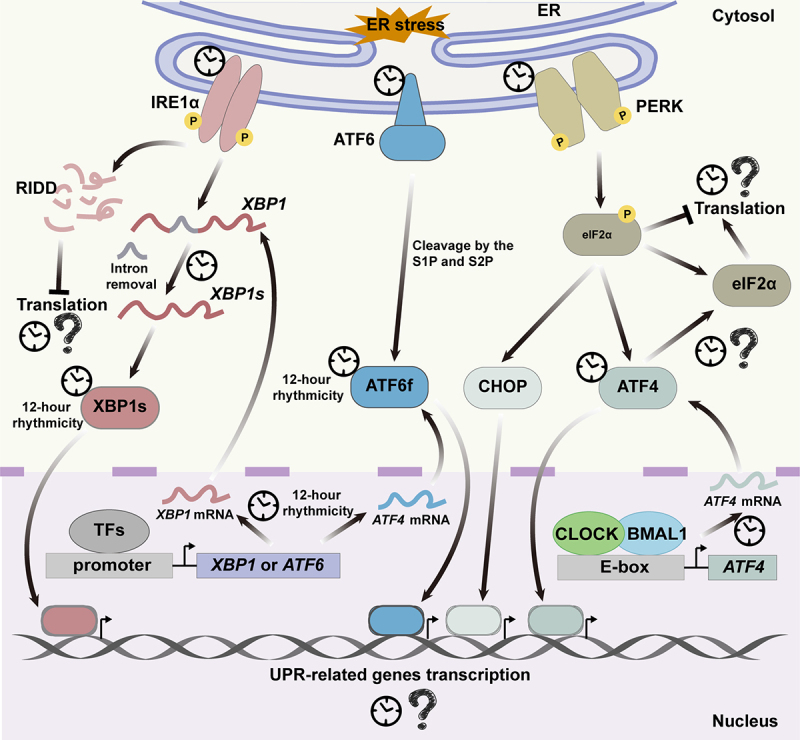
The crosstalk between mRNA translation and ER stress in circadian rhythms. The UPR pathway in response to ER stress consists of three regulatory pathways dominated by transmembrane proteins, namely PERK, IRE1α, and ATF6α. The circadian rhythms of these three pathways have been reported, yet it remains unknown whether the expression of their downstream gene and the inhibition of their overall translation exhibit rhythmicity.

The oscillated expression of numerous proteins associated with ER stress begs the question: do the global translation inhibition during stress and the translation of specific mRNAs essential for adaptive stress response also exhibit rhythmic fluctuations? Here, we summarize current knowledge of how ER stress regulates translation and its involvement in circadian rhythm processes while exploring whether ER stress directly impacts rhythmic protein synthesis (Figure 4).

ER stress triggers the unfolded protein response (UPR) pathway, involving three regulatory pathways primarily controlled by transmembrane proteins: PERK, IRE1α, and ATF6α. Proteins in the PERK pathway play a crucial role in regulating translational processes under endoplasmic reticulum (ER) stress conditions and show rhythmic expression and phosphorylate eIF2α, inhibiting global mRNA translation. PERK undergoes oligomerization and transphosphorylation during ER stress, leading to the phosphorylation of eIF2α and hindering global translation initiation [52]. This helps alleviate protein overload in the stressed ER and activates the translation of specific mRNAs, including ATF4 and CHOP. ATF4, a central transcription factor in the PERK pathway, is subject to Clock-mediated transcriptional regulation, establishing a link between the PERK pathway and circadian expression patterns [53]. CHOP also exhibits rhythmic activation in response to ER stress, and both ATF4 and CHOP work together to promote increased expression of their target genes [54]. The rhythmic expression of the two transcription factors may impact downstream UPR-related processes through oscillations in transcription and translation.

On the other hand, the IRE1α pathway, which plays a pivotal role in the endoplasmic reticulum (ER) stress response, is also subject to regulation by circadian rhythm. IRE1α undergoes dimerization and autophosphorylation in response to ER stress, and its phosphorylation exhibits oscillatory dynamics [55]. Following phosphorylation, IRE1α catalyses the excision of the 26nt intron in *XBP1* mRNA, resulting in the generation of an active transcription factor known as XBP1s [56,57]. In mouse liver, IRE1α-mediated *XBP1* splicing exhibits a 12-hour rhythmic cycle and is partially regulated by the circadian clock [55]. During ER stress, XBP1 increases gene expression related to protein folding, secretion, protein translocation to the ER, and lipid synthesis [58]. The RNase domain of IRE1α also regulates RNA stability through regulated IRE1-dependent decay (RIDD) reactions [59]. Although evidence has demonstrated rhythmic regulation of the IRE1α pathway and expression in numerous proteins within this pathway, further verification is required.

In addition, ATF6α, as another pivotal protein in the UPR pathway, exhibits a circadian oscillation that regulates the transcription of UPR-related genes. ATF6α undergoes cleavage, resulting in the release of its cytoplasmic domain (ATF6f), which upregulates the transcription of specific UPR genes [60]. The light stimulation impacts the transcriptional activation of ATF6 downstream target genes, and under daytime restricted conditions, these ATF6 target genes exhibit a 24-hour periodicity of transcription [61,62]. Similar to the IRE1α pathway, there is currently no direct evidence elucidating the impact of rhythmically expressed ATF6 on translational synthesis rhythmicity.

In conclusion, there is plenty of evidence supporting the direct regulation of ER stress responses by circadian rhythms. However, further research is needed to determine if there is a rhythmic inhibition of ER stress on global cellular translation. Rhythmic translation requires specific time-driven activation and subsequent inhibition for restoration to a lower level. Therefore, it is suggested that ER stress may play a key role in linking rhythmic translation activation and inhibition to maintain a normal cycle within cells.

### 5.2. Translational dynamics and the aging of circadian function

In addition to ER stress, cells must also face ageing, which constitutes a chronic stress factor that increases with time. As a result of such age-related stress, the system controlling circadian rhythms becomes less efficient over time, thereby negatively affecting various biological processes, such as rhythmic mRNA translation. Proteomic studies have revealed global alterations in protein levels during brain ageing, particularly showing that genes related to translation exhibit strong decoupling between their proteome and transcriptome with age [63]. The decline in protein synthesis during ageing is a universally observed phenomenon across different organisms [64]. Therefore, it is important to explore the regulation of rhythmic protein synthesis during ageing to understand the mechanisms underlying the ageing process. In this section, we highlight the relationship between ageing and abnormalities in translation initiation and elongation, thus raising awareness about translational changes contributing to disturbances in the circadian rhythm during ageing.

Age has brought significant changes to translation initiation machines. In instance, diseases characterized by premature or accelerated ageing, such as Hutchinson-Gilford progeria syndrome, leads to an increase in ribosome biogenesis in human fibroblast cells [65]. Besides, altering translation also manipulates the ageing process. In yeast, it was shown that the deletion of ribosomal proteins like RpL10 and RpL18 significantly extends the lifespan [63]. Additionally, some initiation factors in protein synthesis have been identified as important regulators of ageing. In nematodes, downregulating the expression of crucial initiation factors (e.g. eIF1, eIF3, eIF4A, and eIF4G) could extend lifespan by up to 50% [66].

In addition, ageing also has a significant effect on translation elongation. The level of translation elongation factor eEF1A1 shows age-dependent changes in glial cells, suggesting its involvement in ageing mechanisms [67]. Activation of eEF2K leads to a slower translation process with improved accuracy, contributing to an extended lifespan [68]. Given the rhythmic expression and regulatory role of translation machinery components in circadian rhythms, age-related alterations in their expression patterns are likely to exacerbate rhythm disorders, leading to abnormalities in ribosome assembly and protein synthesis within aged neurons.

Together, the rhythmic translation is disrupted or altered during ageing due to aberrant expressions of ribosomal proteins, translation initiation factors, and elongation factors. The discrepancy between the transcriptome and proteome during ageing suggests that post-transcriptional regulation may play a crucial role in adapting to ageing. Current research primarily focuses on RNA fluctuations using transcriptomics after ageing, but it is important to pay closer attention to post-transcriptional regulation for a comprehensive understanding of age-related rhythmic alterations.

## 6. Conclusion and perspectives

For organisms on Earth, circadian rhythms play a pivotal role in orchestrating daily activities within a 24-hour timeframe. The regulation of circadian rhythm is commonly believed to be mediated by diurnal oscillations in mRNA and protein levels. The intricate interplay between circadian rhythms and mRNA translation has been demonstrated as indispensable for the generation and maintenance of the 24-hour cycle. In this review, we discussed the mechanism and regulation of rhythmic translation, encompassing aspects such as translation initiation, elongation, phase separation in translation, ER stress-induced translation, and aberrant translation during the ageing clock. Furthermore, we provided an overview of recent advances in understanding how circadian regulation influences cellular biochemical reactions, including the modulatory impact of phase separation on translation rhythm. Besides, it is important to note that this study mainly focuses on the regulation of rhythmic translation in mammals, especially in mouse. Different species exhibit significant variations in circadian rhythms [69]. For example, humans are diurnal, being active during the day and resting at night, while mice are nocturnal, being more active at night. Non-mammalian organisms have different TTFL mechanisms, resulting in distinct rhythms and regulatory processes [69].

In addition, significant challenges remain in fully comprehending the coordinated regulation of circadian rhythms and translation. The functional understanding of clock proteins discovered thus far remains incomplete. Another challenge is related to the recently established role of phase separation in the circadian rhythm of translation. Previous studies have reported that numerous clock proteins possess the potential for phase separation [70], yet further exploration is required to elucidate how this process facilitates their functionality. Most oscillating proteins and mRNAs do not exhibit circadian rhythms in their nascent RNAs [71], suggesting that post-transcriptional regulation likely plays a role in rhythm regulation through mechanisms, such as RNA binding proteins interactions introducing phase separation concepts. It remains unclear whether non-oscillating proteins undergo circadian regulation through other means, such as rhythmic interactions with partner proteins or rhythmic intracellular translocations. Addressing these issues necessitates novel rationales and techniques including incorporating phase separation concepts and identifying additional rhythmic biogenetic pathways. Future research on circadian regulation should enhance our scientific comprehension of this fundamental process, and also offer opportunities for developing medical treatments related to circadian rhythms.
